# Characterizing ex vivo models for studying lipid metabolism in triple negative breast cancer

**DOI:** 10.1016/j.jlr.2026.101074

**Published:** 2026-06-03

**Authors:** Naruenan Wongreantong, Chloe A.K. White, Yong Jin Lim, Karla C. Williams, Thomas J. Velenosi

**Affiliations:** Faculty of Pharmaceutical Sciences, University of British Columbia, Vancouver, British Columbia, Canada

**Keywords:** lipidomics, triple-negative breast cancer, chick chorioallantoic membrane assay, ex vivo tissue culture, modeling lipid metabolism, triacylglycerols, phospholipids, sphingolipids

## Abstract

Lipid metabolism drives cancer progression but is difficult to model using conventional methods. In vivo models provide access to circulating lipids, but are costly and low throughput, while in vitro models lack sufficient lipid availability in culture media. Here, we evaluate the chick chorioallantoic membrane (CAM) patient-derived xenograft (PDX) and tissue slice models as ex vivo cancer models of lipid metabolism in triple-negative breast cancer (TNBC) over a 9-day incubation period using lipidomics analysis. Differences in the TNBC tumour lipidome were driven by inherent differences between primary and metastatic tumours, then by ex vivo model. Ether-linked phosphatidylcholine and phosphatidylcholine lipids accumulated in CAM xenografts from primary and metastatic TNBC tumours (q < 0.05) but were unchanged in ex vivo tumour slices. Conversely, elevated triacylglycerol, sphingomyelin, and phosphatidylglycerol lipids were prominent in ex vivo tumour slices from primary and metastatic TNBC models when compared to CAM-PDX. Cryopreserved tumour fragments were successfully reanimated, demonstrating a similar lipidome profile to freshly engrafted TNBC tumour fragments in the CAM-PDX model. Hierarchical clustering of total saponifiable fatty acids revealed ex vivo model dependent differences, suggesting that the lipids availability to the tumour environment may influence fatty acyl composition, but not the distribution of esterified lipids. Our findings define the time-dependent lipid subclass accumulation in CAM-PDX and ex vivo tumour slice models, and highlight their utility in the study of the TNBC lipidome.

## Graphical Abstract

**Figure:**
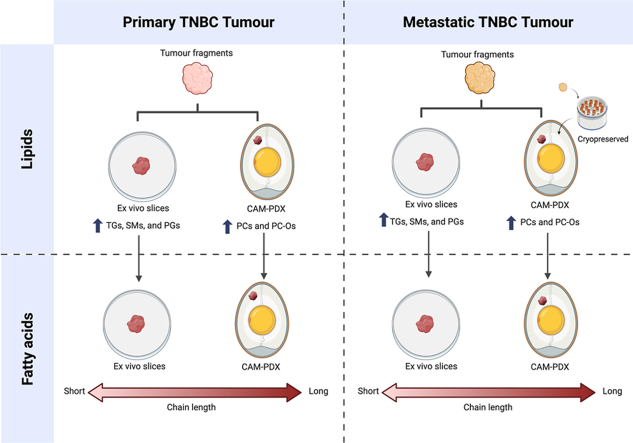

The reprogramming of lipid metabolism is a cancer hallmark (1). Cancer cells require lipids for membrane formation, cell signaling, and a source for fuel (2). Lipids are primarily obtained through the diet or synthesized in the liver by de novo lipogenesis (DNL), then circulated through the body for cell uptake (1, 2, 3). Circulating lipids are the main source of fatty acids for somatic cells, but also feed cancer cells to support growth and proliferation. In addition to circulating lipids, highly proliferative cancer cells reactivate DNL as a secondary mechanism to generate their own FAs (4). Together, the supply of circulating and DNL generated FAs support tumour growth and survival (5).

Breast cancer arises in an adipocyte-rich environment. Elevated lipids in the tumour microenvironment (TME) enhance FA uptake transport into breast cancer cells (6). Triple-negative breast cancer (TNBC) is defined by the absence of drug targets and is the deadliest breast cancer subtype. TNBC prevalence is 10%–20% higher in obese patients than other breast cancer subtypes (7). While upregulation of FASN increases DNL, TNBC tumours also have a high capacity for lipid uptake, which contributes to progression and metastasis (8).

Studying lipid metabolism remains challenging due to the limitations of conventional models (9). Standard in vitro culture media does not contain lipids, requiring cells to acquire lipids from the small percentage of supplemented FBS or by DNL. In addition, tumour progression and treatment response depend on interactions within the TME, which are lost with the use of cell lines or tumour dissociated cells cultured in vitro. In vivo**,** genetically engineered cancer models and xenograft models provide tumours with access to circulating lipids but are low throughput and expensive (10, 11). Therefore, there is an unmet need to develop robust models to study cancer lipid metabolism.

Ex vivo models can bridge the gap between in vitro and in vivo approaches. These models retain tumour structure and cell-cell interactions that exist in the TME in vivo (12, 13, 14). They can also be performed at higher throughput than in vivo approaches. Several studies have evaluated tumour carbohydrate and amino acid metabolism using ex vivo tissue slices, but lipid metabolism has yet to be characterized (12, 15).

The chick chorioallantoic membrane (CAM) xenograft model provides a self-contained and scalable platform for studying cancer lipid metabolism. Angiogenesis stimulated by engrafted cancer cell lines or patient-derived tumours are continuously supplied with circulating lipids provided by the yolk, recapitulating the in vivo vascular network (11, 16). In addition, the CAM xenograft model can support tumour growth for several days allowing for the evaluation and comparison of time-dependent changes in lipid profiles (17). Importantly, the CAM xenograft model can also revive previously frozen tumours, negating the need to engraft fresh patient tumour fragments, which increases the versatility of this model (18).

Here we present an untargeted lipidomics analysis of the ex vivo CAM-xenograft and ex vivo tumour slice models generated using TNBC patient-derived xenografts (PDXs) from primary and metastatic tumours. The objective of this study was to characterize the CAM xenograft and ex vivo tissue slice models for their utility in evaluating TNBC lipid metabolism. Fresh and previously frozen tumour fragments were cultured for nine days to characterize the time-dependent lipid remodeling in each model. Analyses included TNBC tumours derived from primary and metastatic sites to investigate potential differences in lipid metabolism. Notably, the CAM-xenograft model revealed a progressive accumulation of specific lipids that were absent in ex vivo cultured slices, which included differences between primary and metastatic tumours. The findings highlight the individual utility of CAM-xenograft and ex vivo tumours slice models as a robust and cost-effective approaches for studying the TNBC lipidome.

## Material and methods

### Animal models

TNBC-PDX tumours and female NSG mice were obtained from Jackson Labs. Primary TNBC (TM00099, P9, BR1367F, Invasive Ductal Carcinoma, AJCC IIA/Grade:3) and metastatic (TM00096, P18, BR0851F, Invasive Ductal Carcinoma, AJCC IIA/Grade:3) tumours were engrafted into 5–8 week old NSG mice. When TNBC tumours reached 1000–1500 mm^3^, mice were euthanized and tumours excised. Fresh tumour fragments were flash frozen in liquid nitrogen, cryopreserved, engrafted onto chick embryo CAMs, or sliced into viable ex vivo tissue slices. All animal experiments were approved by the University of British Columbia Animal Care Committee (#A21-0195).

### Ex vivo tumours slice preparation

TNBC-PDX tumour bearing mice were euthanized, and tumours were excised aseptically. Tumours were cut in half along the major axis. One half was placed, flat side down, on a tissue chopper slide, covered in 2% low melting point agarose and sliced into 500 μm slices using a McIlwain Tissue Chopper (Ted Pella, Inc.), as previously described (12). TNBC tumour slices were washed twice in PBS and placed into a 6-well plate containing 3 ml of prewarmed RPMI (Corning) containing 10% FBS and 100U/ml penicillin/streptomycin/antimycotic (corning). Ex vivo slices were cultured on an orbital shaker in a humidified 37°C incubator under 5% CO_2_. Fresh media was provided to ex vivo slices every two days and slices were harvested on day 2, 4, 7, and 9 for analysis.

### CAM-PDX preparation

The remaining half of the excised TNBC-PDX tumours from above was segmented into ∼2 mm^3^ fragments and used for CAM-PDX engraftment. Fertilized eggs were obtained from Poultry Research Centre (AB, Canada). The eggs were incubated for 2 days at 37.8°C with rotation. On embryonic day 3, the eggs were cracked, placed into individual weigh boats with lids, and incubated for an additional 7 days at 37.8°C in a humidified incubator (19). On embryonic day 9.5, the surface of the CAM was scored and tumour fragment engraftment. Tumour engraftments were performed using fresh tumour and cryopreserved tumour fragments. Cryopreserved tumour fragments were slowly frozen to −80°C in 90% FBS and 10% DMSO then stored in liquid nitrogen vapor. Tumour samples were collected over the engraftment period at day 2, 4, 7, and 9 postengraftment for analysis. Biological replicates (n per group) were defined as individual chick embryos with successful tumour engraftment (20).

### Lipidomics analysis

#### Sample preparation

Lipids were extracted from frozen tumour fragments and slices using a modified Matyash method (21, 22). Briefly, samples were suspended in ice-cold Milli-Q water and methanol (30:225, v/v) at a ratio of 1 mg per 60 μl. Samples were homogenized in bead-mill homogenizer for 2 cycles of 30s at 6.5 m/s, using zirconium beads (BioSpec). 750 μl of ice-cold methyl tert-butyl ether (MTBE) containing 0.5 μM C17-ceramide (extraction internal standard) was subsequently added to 255 μl of homogenate. The samples were vortexed and incubated for 10 min (4°C, 650 rpm). 188 μl of Milli-Q water was added to followed by centrifugation for 20 min (16,000 × *g*, 4°C). 300 μl of MTBE-containing layer was pipetted into a glass tube and evaporated under nitrogen (TurboVap). Extracted lipids were reconstituted in 200 μl of isopropanol: acetonitrile (50:50, v/v) for LC-MS analysis. A pooled sample was prepared as a quality control (QC) by combining 5 μl from each sample. Samples were maintained at 12°C and were randomized. Pooled samples were injected every 10 samples.

### Saponification

A second 102 μl volume of homogenate was aliquoted, and lipids were extracted using 300 μl of ice-cold MTBE containing 5 μg/ml TG(17:0/17:0/17:0)-d5 (extraction internal standard), as described above. A 200 μl volume of the MTBE (top layer) was evaporated under nitrogen and lipids were saponified in 1 ml of 90/10 MeOH/H_2_O (0.3 M KOH) for 1 h in a water bath at 80°C (23). Saponification was terminated with the addition of formic acid and lipids were extracted with 1 ml of hexane. The extraction was repeated twice, and the samples were dried under nitrogen then resuspended in 100 μl isopropanol/ACN (50:50 v/v) for LCMS analysis.

### Data acquisition

The LCMS method is previously described (22). Briefly, a 2 μl volume of each sample was injected onto an ACQUITY Premier CSH C18 column (2.1 × 100 mm, 1.7 μm particle size) maintained at 65°C in a Thermo Scientific Vanquish UHPLC system. Samples were kept at 16°C in the autosampler. The mobile phase A consisted of 60% acetonitrile, 38.9% water, 0.1% formic acid, and 1 M ammonium formate and mobile phase B consisted of 88.9% 2-propanol, 10% acetonitrile, 0.1% formic acid, and 1M ammonium formate. The flow rate was set to 0.250 ml/min and the following gradient was used: 0–1 min, 15% B; 1–2 min, 15%–30% B; 3–3.5 min, 30%–48% B; 3.5–12 min, 48%–82% B; 12–13 min, 82%–99% B; 13–13.1 min, 99%–15% B; 13.1–17 min, 15% B. All gradients were linear. Total run time was 17 min.

Mass spectrometry analysis was conducted using Thermo Scientific Orbitrap IQ-X Tribid Mass Spectrometer. Electrospray ionization followed the following source parameters were used: 3.5 kV spray voltage for positive ion mode and 2.4k for negative ion mode, 300°C ion transfer tube, 350°C vaporizer temperature. Ions in MS1 were acquired between 250–1500 m/z in the orbitrap (120k resolution at 200 m/z) and the maximum injection time was set to 246 ms. The AGC target was 4e5 and RF Lens% was set to 30%. Data-dependent acquisition was performed using the AcquireX workflow to generate fragmentation spectra from pooled samples. MS2 spectra were acquired at 30 k resolution with an AGC target of 5e4 and a maximum injection time of 54 ms. The minimum intensity threshold for precursor selection was set to 2.5e4. Stepped higher-energy collisional dissociation energies of 20%, 30%, and 40% were alternated with 30% collision-induced dissociation to generate MS2 fragment ions. Positive and negative mode ionization was acquired in separate injections. Total saponifiable fatty acids were acquired in negative ionization mode using the same LCMS method using a m/z range of 100–1000.

### Data analysis

Raw data were processed in Compound Discoverer 3.3 (Thermo Fisher Scientific). Compound Discoverer performed peak integration, retention time alignment, feature detection and grouping, SERRF QC correction, and compound identification using built-in spectral libraries, including LipidBlast (VS 68) and mzCloud (2021B) (Supplemental Table S1). Positive and negative ion mode data were processed separately. The level of lipid annotation was determined using the Lipid MAPS classification system (24). The data was archived and exported for statistical analysis in R (version 4.4.1). Total saponifiable fatty acids were analyzed using Skyline (version 24.1.1.254) and normalized to the total sum area of all fatty acids in each sample.

Features with relative standard deviation (RSD) in QC samples greater than 30% were excluded prior to statistical analysis. Lipid features were normalized to total lipids. Positive and negative ion mode data were combined, as previously described (25). Briefly, lipid features that ionize in positive and negative mode were determined by retention time alignment and neutral mass threshold 5 ppm. Matched features with an abundance correlation of > 0.6 were filtered by choosing the more sensitive ion.

### Statistical analysis

Data were mean centered and pareto scaled. Principal component analysis (PCA) and partial-least squares discriminant analysis (PLS-DA) were conducted using stats (3.6.2) and mixOmics (6.28.0), respectively, in R. PLS-DA validation was performed by k-folds cross validation using 5 folds and 10 repeats (Supplemental Fig. S9). Univariate statistical analysis was performed using Kruskal-Wallis and Dunn's test post hoc test (stats 3.6.2 & FSA 0.9.5) followed by Benjamini-Hochberg false discovery rate correction. Features with q < 0.05 were considered statistically significant. Significantly altered features in CAM and tissue slice samples were normalized to fresh-frozen tissue within each tumour model, then further analyzed by divisive analysis algorithm (DIANA) clustering to group lipids by longitudinal patterns (DEGreport 1.40.1).

Total saponifiable fatty acids are presented the relative change during the incubation period using a ratio of day 7 to day 2. The heatmap was generated using ComplexHeatmap (version 2.20.0). Similarities between total saponifiable fatty acids between samples were determined by hierarchical clustering analysis (version 4.4.1).

## Results

### Primary and metastatic TNBC tumours have distinct lipidomes

**Fig. 1:**
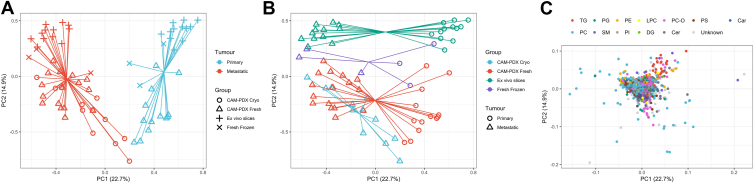
PCA analysis of primary and metastatic tumours. Principal component analysis (PCA) scores plot (A and B) revealed inherent lipid differences between primary and metastatic tumours and tumour samples derived from different ex vivo models, respectively. The loadings plot (C) demonstrates separation driven by lipid species, identified to a minimum of species level identification. PCA, principal component analysis.

In total, 9757 putative lipid features were detected. Interestingly, inherent lipid differences between primary and metastatic tumours superseded methods of tumour harvesting, engraftment or culturing (Fig. 1A, B). PCA separation was driven by elevated phosphatidylcholine (PC), ether phospholipids (PC-O), and triacylglycerol (TG) species in the primary TNBC, and by PC species in the metastatic TNBC samples (Fig. 1C). QC samples clustered together in the center of the PCA plot (Supplemental Fig. S1). The C17-ceramide extraction precision across all samples was 10.8% RSD. To investigate the difference between ex vivo incubation methods over time, the primary and metastatic tumours were evaluated separately.

### Lipid accumulation in primary TNBC depends on the ex vivo model

**Figure 2:**
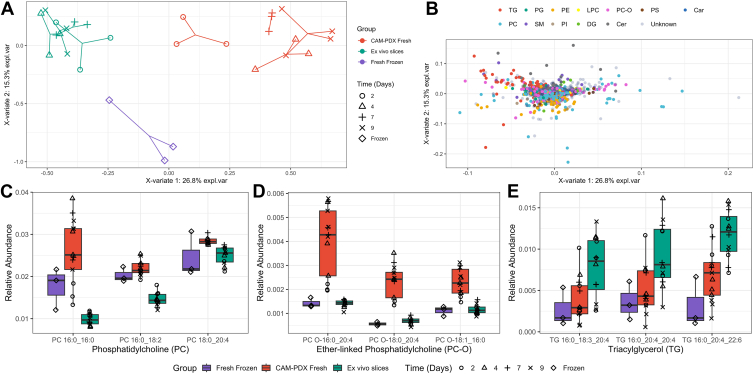
Ex vivo environment drives lipidome differences in primary TNBC. A: PLS-DA scores plot showed lipid profile differences primarily driven by engraftment or culturing period, followed by differences between tumour samples derived from ex vivo models and fresh frozen tumours from mice. Data shown represent n = 3–5 for each ex vivo model at each time point. B: PLS-DA loadings plot showed lipid features driving the lipidome differences, identified to a minimum of species level identification. Top loadings showed elevated PC (C) and PC-O (D) molecular species in CAM-PDX samples compared to ex vivo samples and elevated TGs molecular species (E) in ex vivo samples. CAM, chick chorioallantoic membrane; PC, phosphatidylcholine; PC-O, ether phospholipids; PLS-DA, partial-least squares discriminant analysis; PDX, patient-derived xenograft; TG, triacylglycerol; TNBC, triple-negative breast cancer.

In the primary tumour, PLS-DA analysis revealed lipid differences mainly driven by engraftment or culturing period, followed by differences between tumour samples derived from mice and both ex vivo models (CAM-PDX and ex vivo slices) (Fig. 2A). Elevated PC and PC-O species were associated with CAM-PDX samples when compared to tumour slice samples (Fig. 2C, D), whereas TGs were elevated in tumour slices (Fig. 2D).

**Fig. 3:**
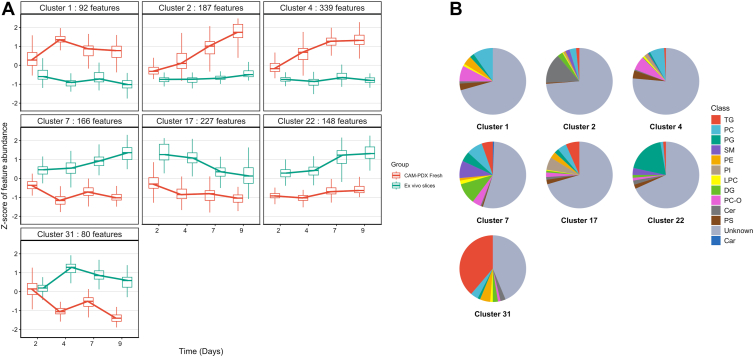
Longitudinal lipidome clustering analysis of primary TNBC in CAM-PDX and ex vivo slice models. A: Lipids exhibiting similar lipid kinetics during CAM-PDX or ex vivo slice incubation are grouped into the same DIANA clusters. Six clusters with distinct kinetics were identified. Data are presented as mean ± SEM, n = 3–5. B: Lipid subclasses corresponding to each cluster, identified to the minimum of species level annotation. Clusters are presented as box plots, which include median, interquartile range (IQR) and 1.5xIQR whiskers. CAM, chick chorioallantoic membrane; DIANA, divisive analysis algorithm; PDX, patient-derived xenograft; TNBC, triple-negative breast cancer.

Notably, lipid profile differences between CAM-PDX and ex vivo slices demonstrated a clear incubation time dependence, suggesting differences in the lipid supply and interaction with the tumour environment. DIANA clustering analysis was performed to evaluate longitudinal lipid changes between CAM-PDX and ex vivo tumour slices. We identified seven clusters with distinct lipid kinetic patterns when comparing CAM-PDX and tumour slices from primary TNBC (Fig. 3). Cluster 1 demonstrated elevated PC and PC-O lipids in the CAM-PDX models which were sustained over time. Clusters 2 and 4 displayed a time-dependent accumulation of PC, PC-O, and ceramide (Cer) species over the CAM-PDX incubation period, with minimal changes in tumour slices (Fig. 3A, B). Conversely, lipids in clusters 7 and 22 increased in tumour slices over time compared to CAM-PDX. Cluster 22 mainly containing phosphatidylglycerols (PG) while cluster 7 consisted of several PC, sphingomyelins (SM), and diacylglycerols (DG). Clusters 17 and 31 contained more abundant lipids in ex vivo slices that decreased or were unchanged over the incubation period, respectively. These clusters were comprised of various lipid species with TGs being the most prevalent. (Fig. 3A).

### Lipid dynamics in metastatic TNBC are mainly driven by ex vivo model then incubation period

**Fig. 4:**
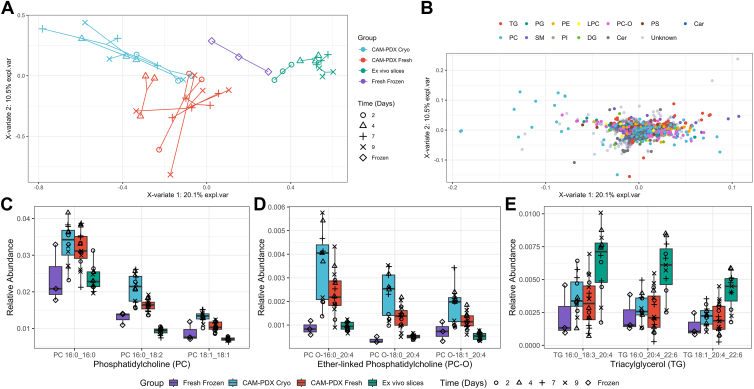
The metastatic TNBC lipidome is mainly affected by ex vivo environment then by preservation method. A: The score plot showing lipidome variation being driven by culturing or engraftment period, followed by tumour samples derived from different ex vivo models and fresh frozen tumours from mice. Data shown represent n = 3–5 for each ex vivo model at each time point. B: The loading plot revealed which lipid features, identified to a minimum of species level identification, driving the variation, with elevated PC (C) and PC-O (D) molecular species in CAM-PDX samples and elevated TG (E) molecular species in ex vivo slices samples. Lipid classes are presented as box and swarm plots, which include median, interquartile range (IQR), 1.5xIQR whiskers as well as individual n values. CAM, chick chorioallantoic membrane; PC, phosphatidylcholine; PC-O, ether phospholipids; PDX, patient-derived xenograft; TG, triacylglycerol; TNBC, triple-negative breast cancer.

In the metastatic TNBC samples, the PLS-DA score plot revealed lipid profile differences primarily driven by ex vivo model (CAM-PDX or tumour slice) (Fig. 4). CAM-PDX and ex vivo tumour slice models diverged from their derived mouse frozen samples along the first latent variable, which accounted for 20.1% of the lipidome variation (Fig. 4A). Elevated levels of select PCs comprised of abundant fatty acyls (16:0, 18:1, 18:2) (Fig. 4C) and PC-Os containing arachidonic acid (20:4) (Fig. 4D) were associated with CAM-PDX samples. Ex vivo tumour slices contained elevated SM, PC, and several TG species (Fig. 4E).

**Fig. 5:**
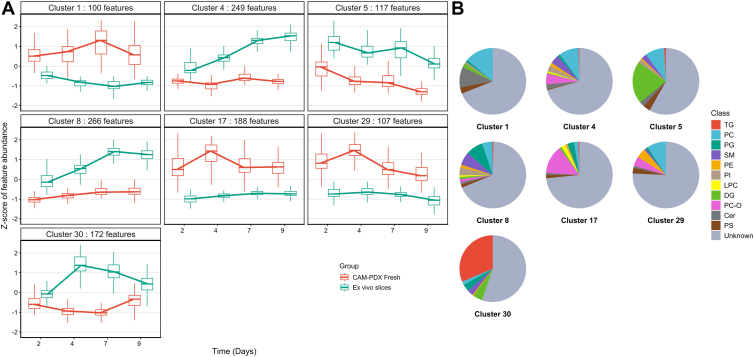
Longitudinal lipidome clustering analysis of metastatic TNBC in CAM-PDX and ex vivo tumour slice models. A: Lipids exhibiting similar kinetics patterns are grouped into the same DIANA clusters. Seven clusters demonstrating distinct kinetics patterns were identified. Data are presented as mean ± SEM, n = 3–5. B: Distribution of lipid subclasses contributing to each cluster, identified to a minimum of species level annotation. Clusters are presented as box plots, which include median, interquartile range (IQR) and 1.5xIQR whiskers. CAM, chick chorioallantoic membrane; DIANA, divisive analysis algorithm; PDX, patient-derived xenograft; TNBC, triple-negative breast cancer.

To directly compare CAM-PDX and tumour slices from freshly isolated TNBC metastatic tumours over time, we performed DIANA clustering analysis. A total of seven clusters exhibiting distinct kinetic patterns were identified (Fig. 5). Clusters 1, 17, and 29 predominantly demonstrated elevated PCs, Cers, and PC-Os, respectively (Fig. 5A, B), in CAM-PDX samples, which were sustained over the incubation period. Cluster 4 and 8 demonstrated elevated levels of various lipid species reaching steady-state by day 7 in ex vivo slices but were unchanged to CAM-PDX samples. Cluster 30 contained TGs as the predominant lipid subclass, which accumulated until day 4 then decreased over time in ex vivo slices. Cluster 5 displayed a time-dependent reduction in DG and PC species in both models, but the levels were consistently higher in ex vivo compared to CAM-PDX samples (Fig. 5)

### Cryopreserved and fresh TNBC tumour fragments demonstrated a similar lipid profile when engrafted into CAM-PDX

Metastatic CAM-PDX and ex vivo slices demonstrated clear divergence in their lipid profiles over time, with many changes evident after just 2 days of incubation. However, cryopreserved and freshly engrafted CAM-PDX samples were remarkably similar after 4 days of incubation despite the additional steps of cryopreservation and thawing (Fig. 4A and Supplemental Fig. S2). Toward the end of the 9-days incubation, notable differences included a greater accumulation of PC-Os and marked decrease in PCs in previously cryopreserved CAM-PDX when compared to freshly engrafted tumour fragments indicating that cryopreservation and revival may have a long-term impact on TNBC lipid metabolism in this model (Supplemental Figs. S2–S4).

### PC and PC-O lipids accumulated in CAM-PDX models when compared to tumour slices

**Fig. 6:**
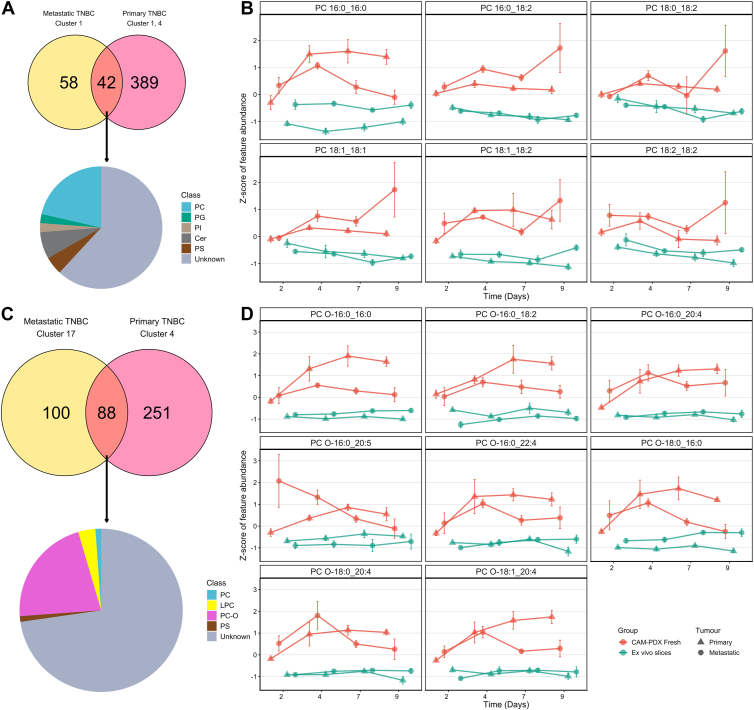
PC and PC-O lipids increased over time in CAM-PDX compared to ex vivo tumour slices. A Venn diagram illustrating the overlap of PC (A) and PC-O (C) lipids demonstrated an increase in CAM-PDX compared to ex vivo tumour slices in TNBC metastatic and primary alongside their lipid class distribution. Representative tracings of PCs (B) and PC-Os (D) molecular species over time which represent the largest identified lipid class from combined primary and metastatic tumours are presented as mean ± SEM, n = 3–5. CAM, chick chorioallantoic membrane; PC, phosphatidylcholine; PC-O, ether phospholipids; PDX, patient-derived xenograft.

To determine whether lipids from CAM-PDX and ex vivo slices shared similar kinetics, we directly compared clusters between primary and metastatic TNBC tumours. Cluster 1 and 4 from primary TNBC samples and cluster 1 from metastatic TNBC samples contained PCs that accumulated in CAM-PDX models when compared to ex vivo slices (Fig. 6A). These included PCs with common acyls (16:0. 18:0, 18:1, 18:2, Fig. 6B). Similarly, metastatic TNBC cluster 17 and primary TNBC cluster 4 shared several PC-O species (Fig. 6C). PC-Os from CAM-PDX samples increased over the incubation period in both the primary and metastatic TNBC but remained largely unchanged in ex vivo tumour slices (Fig. 6D).

### TG, SMs, and PG lipids accumulated in ex vivo tumour slices when compared to CAM-PDX models

**Fig. 7:**
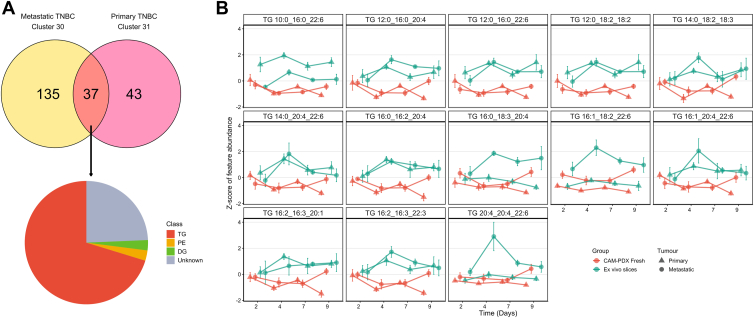
TGs were elevated over the culturing period in ex vivo tumour slices when compared to CAM-fresh. A: A Venn diagram demonstrating the overlap of elevated TG lipids in ex vivo tumour slices compared to CAM-PDX fresh samples in metastatic TNBC cluster 30 and primary TNBC cluster 31. B: Representative tracings of TGs molecular species over time which represent the largest identified lipid class from combined metastatic TNBC cluster 30 and primary TNBC cluster 31 are presented as mean ± SEM, n = 3–5. CAM, chick chorioallantoic membrane; PDX, patient-derived xenograft; TG, triacylglycerol; TNBC, triple-negative breast cancer.

Tumour slices in primary and metastatic TNBC models contained an abundance of TGs (Fig. 7) as well as SMs (Supplemental Fig. S5) and PGs (Supplemental Fig. S6) that were sustained over the incubation period. Interestingly, while the majority of the SM lipids showed greater abundance in ex vivo tumour slices, SM 38:2;2O, SM 42:1;2O, and SM 42:2;2O were more prominent in primary TNBC tumours engrafted onto the CAM compared to ex vivo tumour slices. DGs were also elevated in tumour slices when compared to CAM-PDX samples, but different between primary and metastatic models (Supplemental Fig. S7).

### TNBC tumours accumulate long chain fatty acids during CAM-PDX incubation

**Fig. 8:**
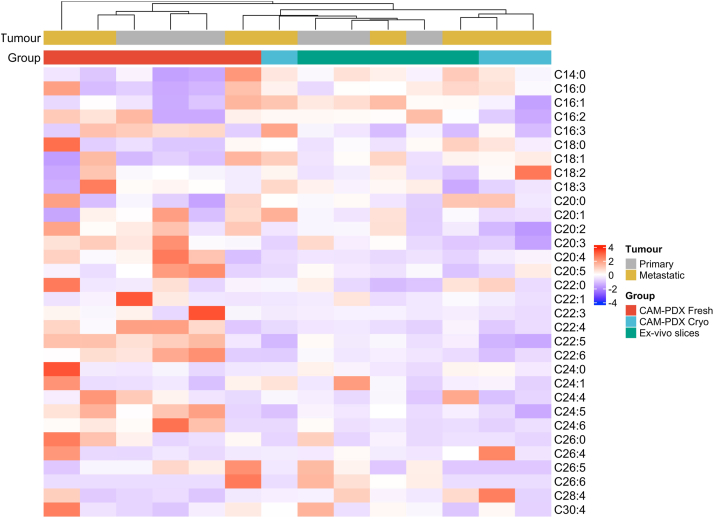
Hierarchical clustering analysis of total saponifiable fatty acids highlights the separation by ex vivo environment. The relative changes in total saponifiable fatty acid abundance between day 7 and day 2 samples cluster by ex vivo model rather than tumour model. Data are presented as the day 7 to day 2 ratio of individual fatty acid abundance for each tumour model and ex vivo model combination.

To determine whether the tumour environment and engraftment method effect fatty acid composition over the incubation period, lipid extracts were saponified and the change in fatty acid distribution between day 7 to day 2 was evaluated. Saponification of the extraction internal standard TG(17:0/17:0/17:0)-d5 across all samples was 16% RSD. Notably, unlike lipidomics analysis, total saponifiable fatty acid hierarchical clustering analysis primarily grouped samples by ex vivo model rather than primary or metastatic tumour model (Fig. 8). CAM-PDX samples accumulated longer chain fatty acids (C20-C26) over time compared to ex vivo slices. In addition, the accumulation of long-chain fatty acids was observed in fresh, but not cryopreserved TNBC tumour samples engrafted onto the CAM.

## Discussion

Cancer cells rely on a combination of DNL and lipid uptake from the TME to sustain their metabolic need for lipids. Studying cancer lipid metabolism can be challenging to model in vitro due to the limited access to extracellular lipids, and in vivo methods are expensive and low-throughout. Here, we provide a untargeted analysis of the ex vivo CAM and tissue slice models for the evaluation of cancer lipid metabolism in TNBC. Unlike cell lines, ex vivo tumour models retain their 3D structure. In addition, cell line models lack the genetic and molecular heterogeneity recapitulated by patient-derived tumours (26).

TNBC tumour samples derived from primary and metastatic TNBC had significantly different lipid profiles, regardless of culturing method. TNBC is a highly heterogenous disease and the baseline differences clearly illustrate inherent heterogeneity between TNBC tumours, which were maintained by ex vivo models.

Lipid profiles in CAM-PDX and ex vivo models diverged from fresh-frozen tumour tissue over the incubation time course. The time-dependent trajectory of the lipidome in CAM-PDX and ex vivo models demonstrate the tumour environment influence of the CAM and tissue culture media, respectively, on tumour lipid metabolism. In CAM-PDX samples, TNBC cells gain access to lipids through vascular circulation from the chick embryo. Primary and metastatic TNBC engrafted onto CAMs were defined by elevated PCs and PC-Os when compared to ex vivo slices and fresh-frozen TNBC-PDX samples derived from mice. Lipids in the yolk provide the chick embryo with ∼90% of its energy requirements (27). The yolk contains mostly TG and PC lipids, which supply the chick embryo and the engrafted TNBC tumour in the CAM-PDX model. Palmitic acid (16:0) and oleic acid (18:1) each account for ∼35% of fatty acid content in the yolk, followed by linoleic (18:2, ∼11%), stearic (18:0, 11%) and arachidonic acid (20:4, ∼2.5%) (28). PC and PC-O lipids found to accumulate in primary and metastatic TNBC tumours supported by the CAM-PDX model mainly contained these fatty acids, suggesting the utilizations of circulating lipids in this model.

Most tissue culture media is supplemented with 10% FBS, which contains the total sum of accessible lipids to support the cultured tissue. Compared to human serum, FBS has 75% less lipid content, including a ∼90% reduction in monounsaturated and polyunsaturated fatty acids (29). Furthermore, at a concentration of only 10% FBS, cultured cells or tissue have limited access to lipids. Most esterified lipids in FBS are from sterol esters and PEs (30), which differs greatly from the CAM-PDX model. In this study, ex vivo tumour slices accumulated more TGs than CAM-PDX suggesting elevated uptake and repackaging of lipids for storage. In addition, the supraphysiological levels of glucose and glutamine in tissue culture media contribute to acetyl-CoA production. When combined with elevated FASN function in TNBC, increased DNL may drive elevated TG production and storage in ex vivo tissue slices (31). In contrast, glucose levels are generally depleted in the TME (32), which may further explain elevated TGs in ex vivo tissue slices when compared to fresh-frozen tumours and CAM-PDX engrafted tumours. Interestingly, DGs also demonstrated a time-dependent increase in ex vivo culture samples compared to CAM-PDX. DG lipids are an intermediate step in TG production (3). This time-dependent increase in DGs further supports the upregulation of TG production under ex vivo culture conditions.

Utilizing freshly acquired patient tumour samples to generate PDX models poses significant logistical challenges. Reduced tumour viability can compromise model engraftment success when generating PDX models (33). Cryopreservation can be used as a method to delay the use of previously acquired TNBC samples for PDX models (34). However, the process of cryopreservation and reanimation of tumour samples may potentially alter tumour lipid metabolism. In this study, we directly compared freshly engrafted and previously cryopreserved TNBC samples using the CAM-PDX model. Freshly engrafted and reanimated TNBC tumour samples demonstrated remarkably similar esterified lipid profiles in CAM-PDX models while total saponifiable fatty acid composition differed. These data suggest that the CAM-PDX model can reliably support lipid metabolism studies using cryopreserved tumour samples when evaluating the esterified lipidome, enabling retrospective clinical investigations that would otherwise be constrained by the need for freshly resected tumour tissue. Unlike mouse PDX models, where time to tumour formation and tumour growth are delayed following cryopreservation (35), our study demonstrates that reanimated tumour samples can be assessed in the 9 days following reanimation and CAM engraftment.

Cancer cells acquire much of their lipids from the surrounding environment (1, 3). Therefore, the model used to support tumour growth may directly impact the tumour lipidome. In this study, the lipid differences between samples were mainly driven by inherent differences between primary and metastatic tumours; however, the fatty acyl composition was shaped by ex vivo model. These data suggest that the TNBC tumours generate lipids according to their needs, but the composition of their fatty acyls depends on the availability of lipids in the TME.

Our study has several strengths and weaknesses. We chose to evaluate the usage of high-throughput and inexpensive ex vivo models that can evaluate patient-derived tumours over several days while reducing the number of mice needed per experiment. In addition, we utilized TNBC tumours derived from primary and metastatic TNBC as well as two ex vivo models to demonstrate the relative contribution of tumour heterogeneity and TME toward the lipidome. Furthermore, we demonstrate the use of cryopreserved then reanimated TNBC tumour fragments in the CAM-PDX model as a method to study patient-derived tumour lipid metabolism. However, we acknowledge that the absence of class-specific internal standards limits our ability to correct the differential matrix effects between the models.

Notably, the reduced accumulation of long-chain fatty acids in reanimated samples may suggest potential alterations in enzymatic activities involved in fatty acid elongation. The potential effects of cryopreservation and reanimation on enzyme activity should be investigated in future studies to determine the utility of cryopreserved models for accurately recapitulating lipid metabolic processes. While this study provides an untargeted analysis of the tumour lipidome in CAM-PDX and ex vivo slice models, further studies are needed to evaluate whether experimental intervention in these models can recapitulate in vivo lipid metabolism. In particular, future isotopic tracing experiments would be valuable to distinguish the extent of lipid metabolism in this model. Future targeted lipidomic analyses would contribute to the overall understanding of these systems and strengthen the overall value of the CAM-PDX and ex vivo slice models. Moreover, given the heterogeneity of TNBC tumours, lipidomics evaluation of additional tumours is needed to capture the TNBC lipidome in these ex vivo models.

In conclusion, our study reveals distinct lipid kinetic patterns observed in CAM-PDX and ex vivo tumour slice models. The CAM xenograft model showed progressive accumulation of PC and PC-O lipids, while the ex vivo tumour slice model displayed elevated TG, SM and PG lipids over a 9-day incubation period. Notably, cryopreserved tumours engrafted onto the CAM xenograft models showed esterified lipid profiles comparable to the freshly engrafted tumours, highlighting the versatility of CAM models for studying the tumour lipidome. This work validates CAM-PDX and tumour tissue slice models and establishes a framework for the high-throughput ex vivo characterization of patient-derived tumour lipidome in TNBC.

## Data availability

Raw lipidomics data, feature lists, and spectra are available from the MetaboLights [31] repository with the study identifier MTBLS13377.

## Supplemental data

This article contains supplemental data(36).

## Conflict of Interest

The authors declare that they have no conflicts of interest with the contents of this article.
