# Supplementary material for: Characterizing ex vivo models for studying lipid metabolism in triple negative breast cancer
Source: J Lipid Res. 2026 Jun 3;67(7):101074. doi: 10.1016/j.jlr.2026.101074 (PMC13329503; doi:10.1016/j.jlr.2026.101074)
Supplement: Supplemental Figures and Tables [file mmc1.docx]

# Supporting Information

**Characterizing ex vivo models for studying lipid metabolism in triple negative breast cancer**

**Naruenan Wongreantong^1^, Chloe A.K. White^1^, Yong Jin Lim^1^, Karla C. Williams^1^, Thomas J. Velenosi^1^**

^1^ Faculty of Pharmaceutical Sciences, University of British Columbia, Vancouver, BC, Canada

**Contents:**

Supplemental Methods

Supplemental References

Supplemental Table S1-2

Supplemental Fig. S1-S11

# Supplementary Methods

**Tumour slices viability assay**

Following the engraftment period, tumours were transferred to fresh medium, and 300 μL of PrestoBlue reagent was added to each sample and incubated at 37 °C for 2 hours (1). Fluorescence intensity (560/590 nm) was measured for Day 0 (fresh), Day 4, and Day 9 samples. Fluorescence values were normalized to tumour weight, and fresh tumours (Day 0) were set as the baseline (100%), with all subsequent values expressed relative to this baseline.

**Quantitative PCR (qPCR) assay**

Human specific primers for fatty acid synthase (FASN) and glyceraldehyde 3-phosphate dehydrogenase (GAPDH) were generated to ensure only human FASN was assessed (**Supplemental Table S2**). RNA extraction was performed on the tumours collected after the viability assay using TRIzol reagent (Quantabio). cDNA was then synthesized using qScript cDNA synthesis kit (Quantabio). The qPCR assay was performed using the PerfeCTa SYBR® Green SuperMix low ROX® (Quantabio) on a QuantStudio 7 Flex (Applied Biosystems). Values are presented as relative expression of FASN calculated using the the 2-∆∆CT method.

# Supplementary References

1. Roife D, Dai B, Kang Y, Perez MVR, Pratt M, Li X, et al. Ex Vivo Testing of Patient-Derived Xenografts Mirrors the Clinical Outcome of Patients with Pancreatic Ductal Adenocarcinoma. Clin Cancer Res. 2016 Dec 15;22(24):6021–30. doi:10.1158/1078-0432.CCR-15-2936 PubMed PMID: 27259561; PubMed Central PMCID: PMC5136340.

# Supplemental Tables

| **Lipid Class** | **Number of Species** |
| --- | --- |
| Cer | 23 |
| DG | 15 |
| HexCer | 21 |
| LNAPE | 10 |
| LPC | 24 |
| PC | 70 |
| PC-O | 41 |
| PE | 26 |
| PE-O | 19 |
| PG | 11 |
| PI | 2 |
| SM | 39 |
| TG | 46 |

**Table S1**. **Number of lipid species identified in each lipid class.** Lipids were identified using Compound Discoverer (3.3) in both positive and negative both mode ionizations. Lipids with identification score less than 40 were excluded.

| **Name** | **Forward or Reverse** | **Sequence** |
| --- | --- | --- |
| FASN | Forward | CAACCTCTCCCAGGTATGCG |
|  | Reverse | CCAGGGAGCTGTGGATGATG |
| GAPDH | Forward | GCTCATTTCCTGGTATGACAACG |
|  | Reverse | GAGATTCAGTGTGGTGGGGG |

**Table S2**. **Primer sequences used for qPCR analysis.** Forward and reverse primer sequences for target gene FASN and the housekeeping gene GAPDH, used to quantify relative gene expression by qPCR.

# Supplemental Figures


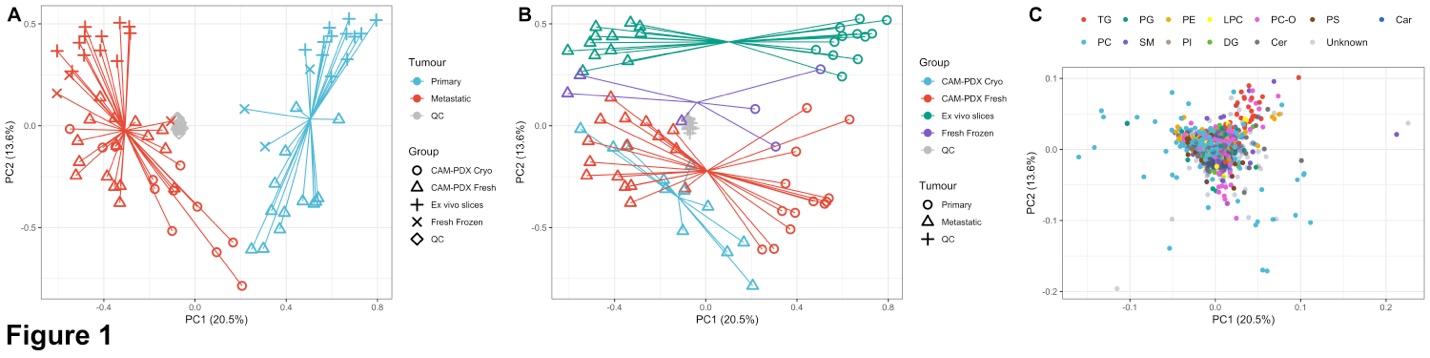


**Figure S1. PCA analysis of primary and metastatic tumours including quality control samples.** Principal component analysis (PCA) scores plot **(A and B)** revealed inherent lipid differences between primary and metastatic tumours and tumour samples derived from different ex vivo models. The loadings plot **(C)** demonstrates separation driven by lipid species. Lipid features shown have been identified to a minimum species level identification.


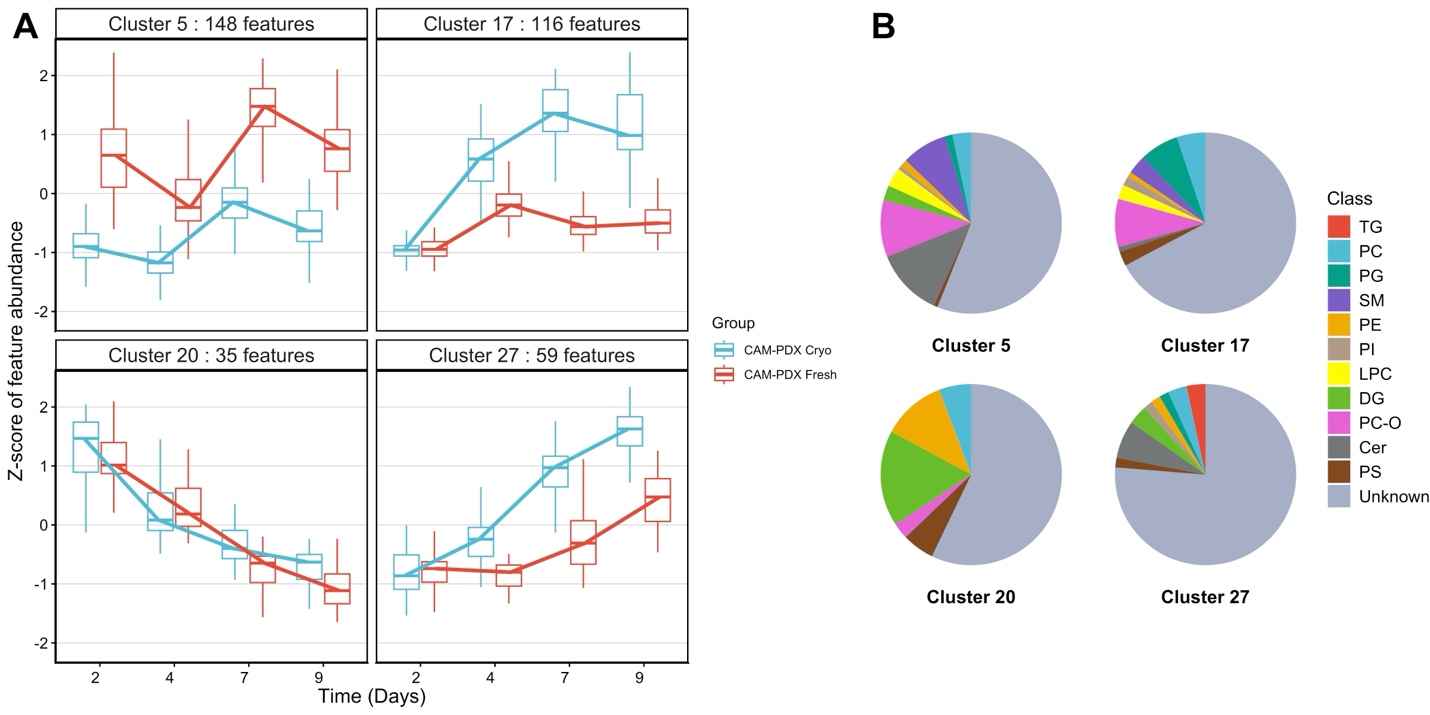


**Figure S2**. **Longitudinal lipidome clustering analysis of metastatic TNBC in CAM-PDX models engrafted with fresh and cryo-preserved TNBC tumours. A)** Lipids exhibiting similar kinetics patterns are grouped by DIANA clustering. Four clusters demonstrating distinct kinetic patterns were identified. **(B)** Distribution of lipid subclasses contributing to each cluster, identified to a minimum of species level annotation. Clusters are presented as box plots, which include median, interquartile range (IQR) and 1.5xIQR whiskers.


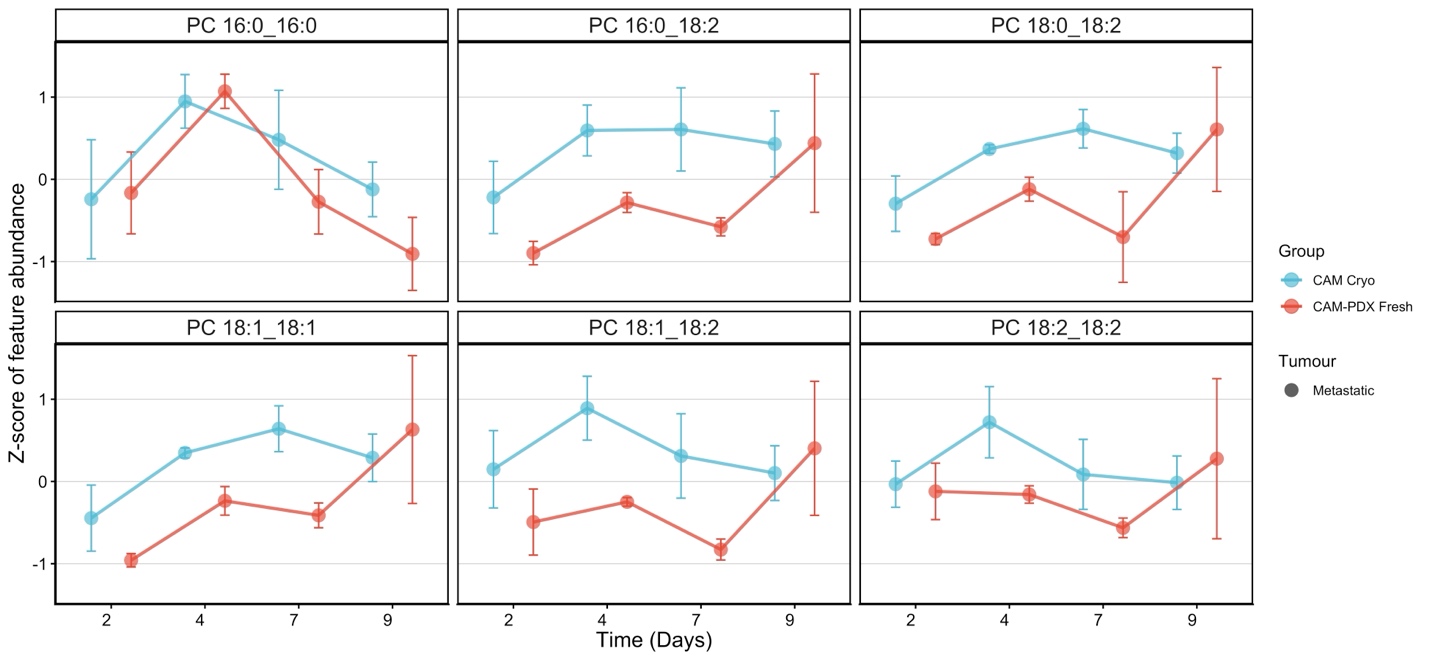


**Figure S3**. **PC lipids exhibited similar lipid kinetic patterns in both fresh and cryopreserved metastatic tumours engrafted onto the CAM-PDX models**. PC lipids, identified to the molecular species level, comprised of abundant fatty acyls (16:0, 18:1, and 18:2) exhibited similar lipidomic profiles in both cryopreserved and fresh TNBC tumours. Representative tracings of PCs over time are presented as mean ± SEM, n = 3-5.


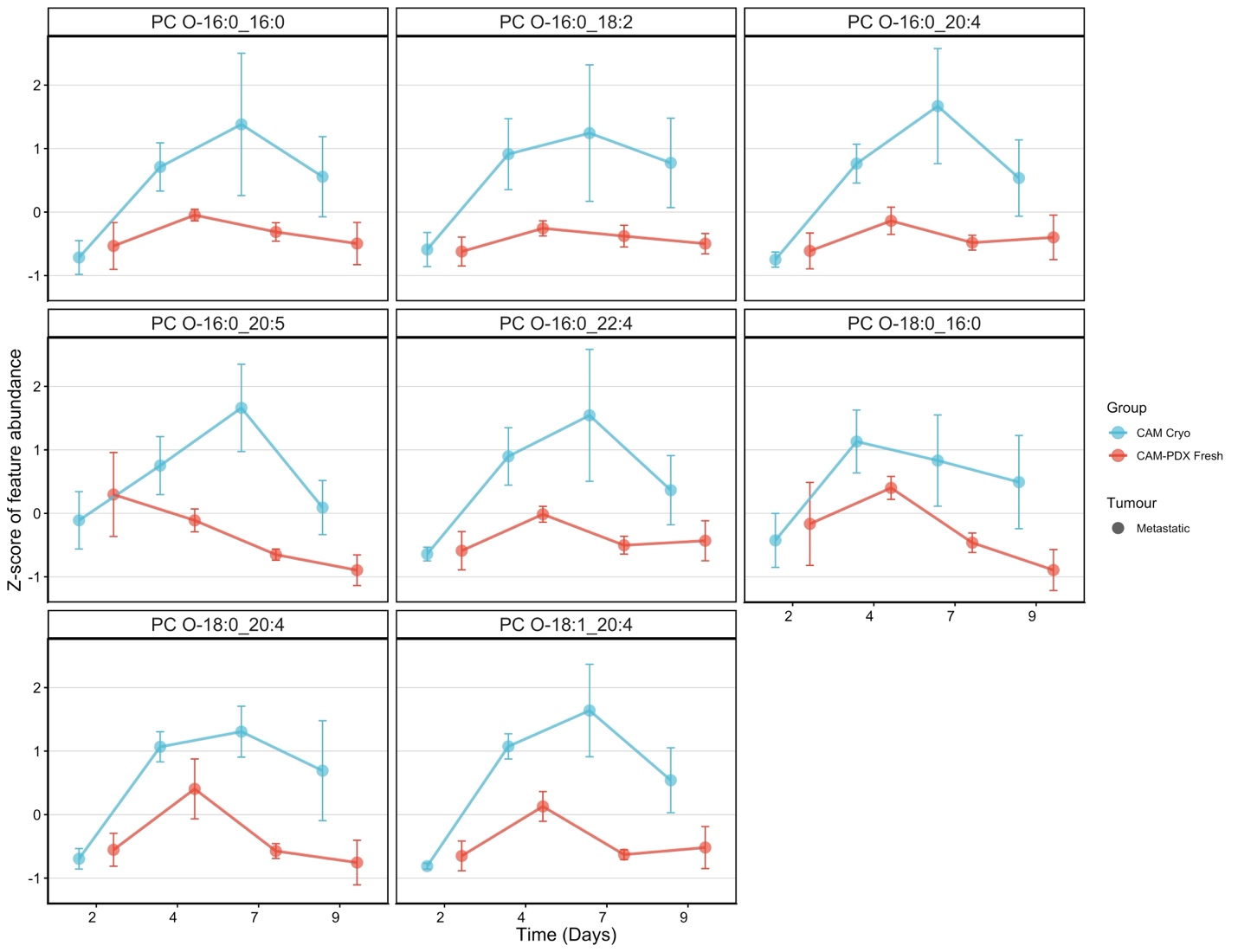


**Figure S4**. **Longitudinal PC-O greater accumulation occurred in cryopreserved tumours compared to freshly engrafted metastatic CAM-PDX.** PC-O lipids, identified to the molecular species level, showed significantly higher accumulation in cryopreserved tumour samples over time. Representative tracings of PC-Os over time are presented as mean ± SEM, n = 3-5.


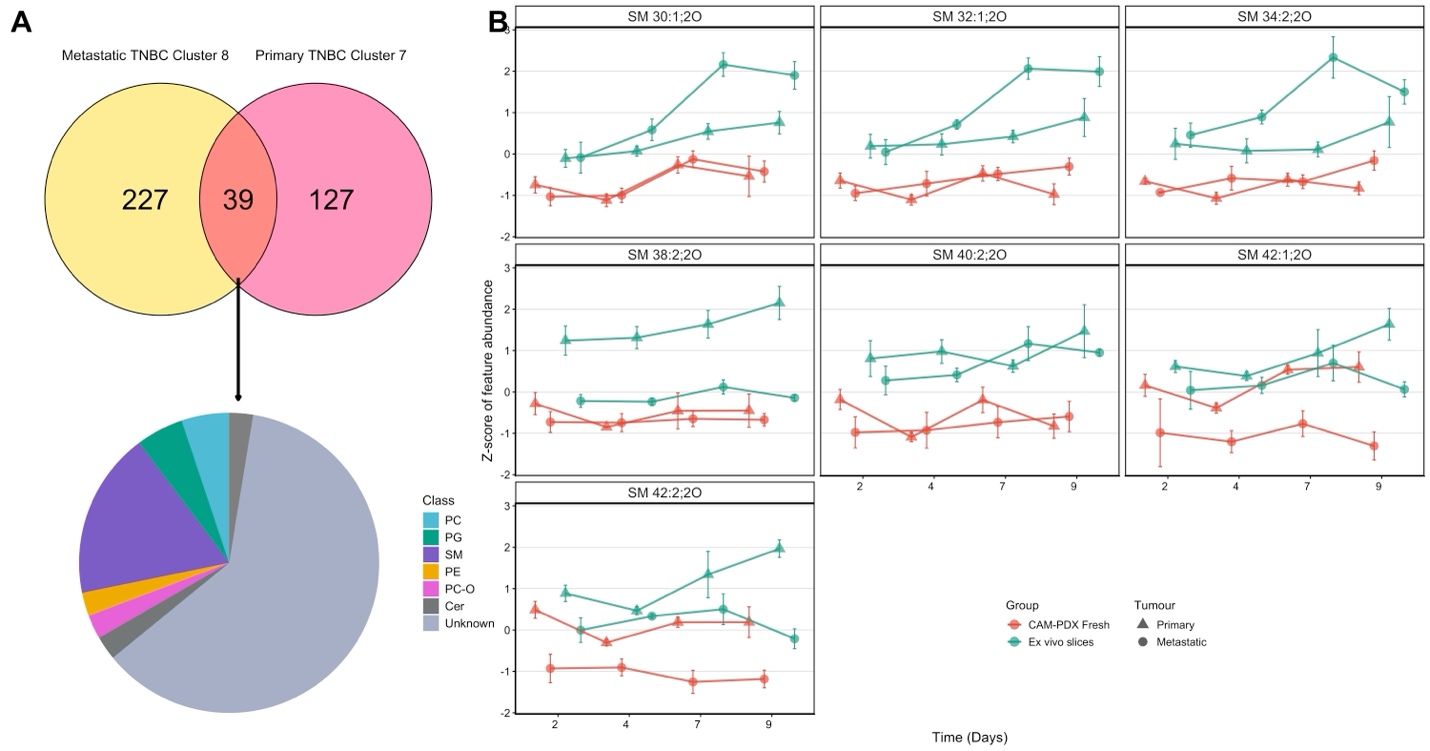


**Figure S5**. **SM lipids were elevated in ex vivo tumour slices compared to CAM-PDX models. (A)** A Venn diagram demonstrating the overlap of elevated SM lipids in ex vivo tumour slices compared to CAM-PDX Fresh samples in metastatic TNBC cluster 8 and primary TNBC cluster 7. (**B)** Representative tracings of SM species over time which represent the largest identified lipid class from combined metastatic TNBC cluster 8 and primary TNBC cluster 7 are presented as mean ± SEM, n =3-5.


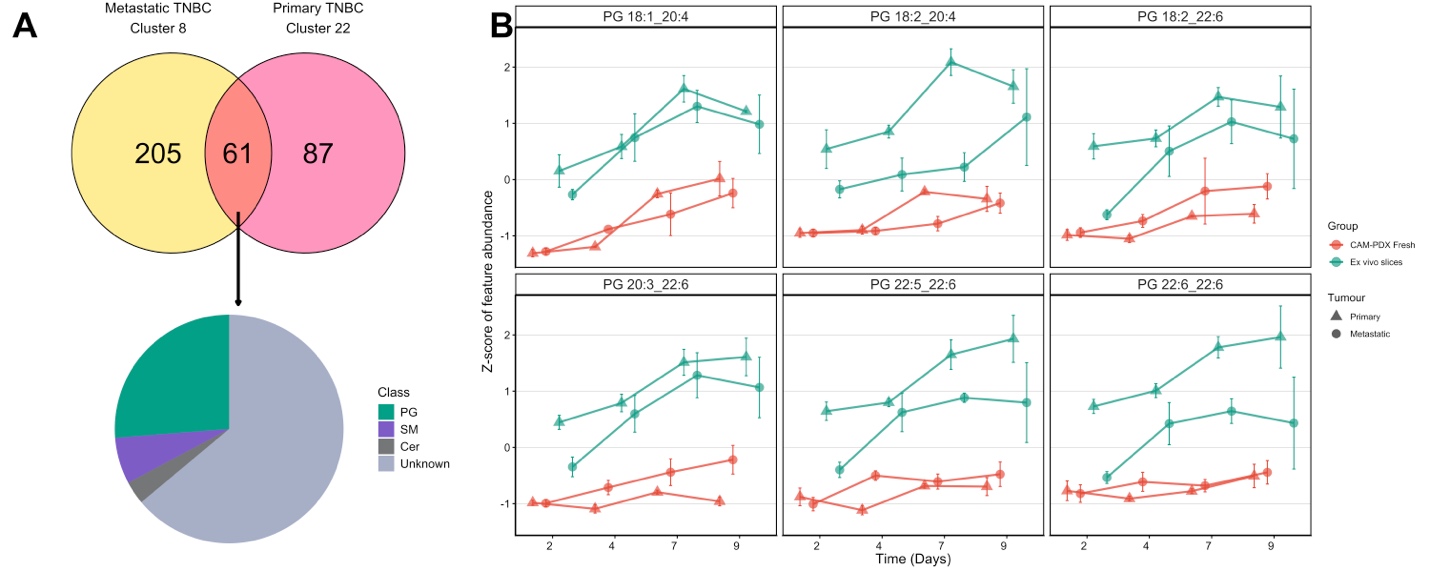


**Figure S6**. **PG lipids were elevated over the culturing period in ex vivo tumour slices compared to CAM-PDX models. (A)** A Venn diagram demonstrating the overlap of elevated PG lipids) in ex vivo tumour slices compared to CAM-PDX Fresh samples in metastatic TNBC cluster 8 and primary TNBC cluster 22. (**B)** Representative tracings of PG molecular species over time which represent the largest identified lipid class from combined metastatic TNBC cluster 8 and primary TNBC cluster 22 are presented as mean ± SEM, n = 3-5.

**
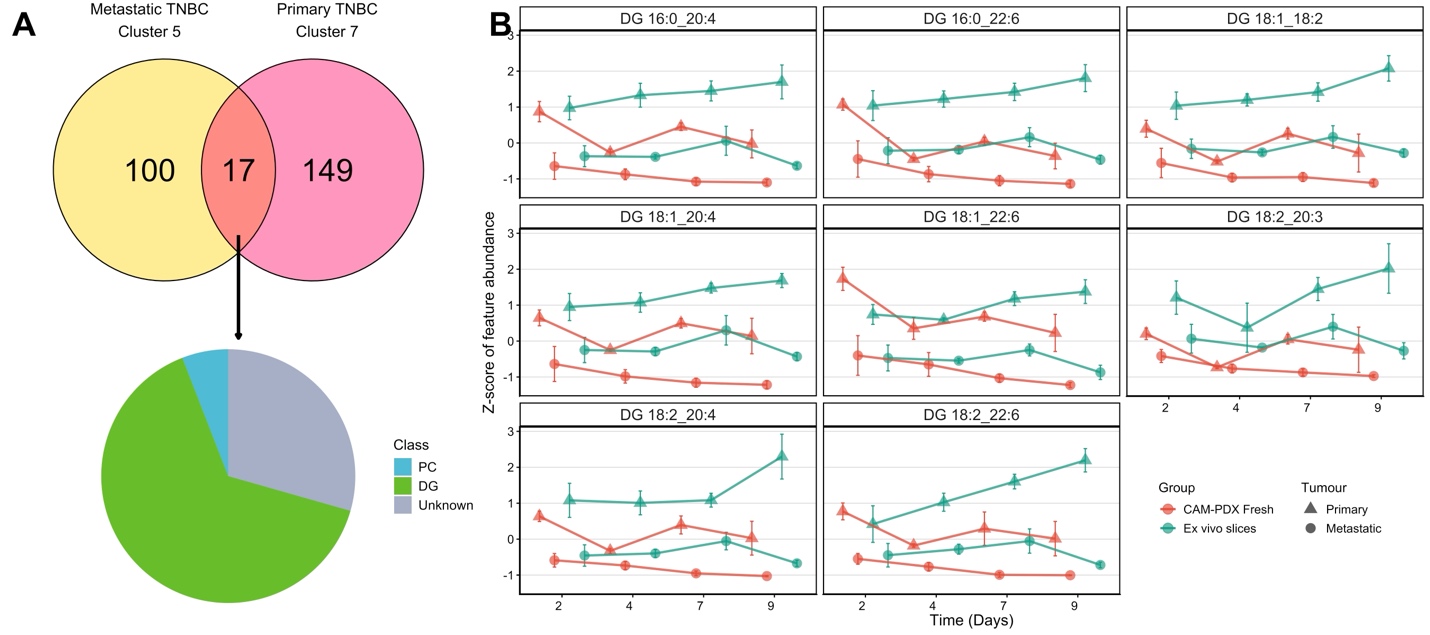
**

**Figure S7**. **DG lipids accumulated in ex vivo tumour slices but not CAM-PDX models over the time course**. **(A)** A Venn diagram demonstrating the overlap of elevated DG lipids in ex vivo tumour slices compared to CAM-PDX Fresh samples in metastatic TNBC cluster 5 and primary TNBC cluster 7. **(B)** Representative tracings of DG molecular species over time which represent the largest lipid species identified from combined metastatic TNBC cluster 5 and primary TNBC cluster 7 are presented as mean ± SEM, n = 3-5.


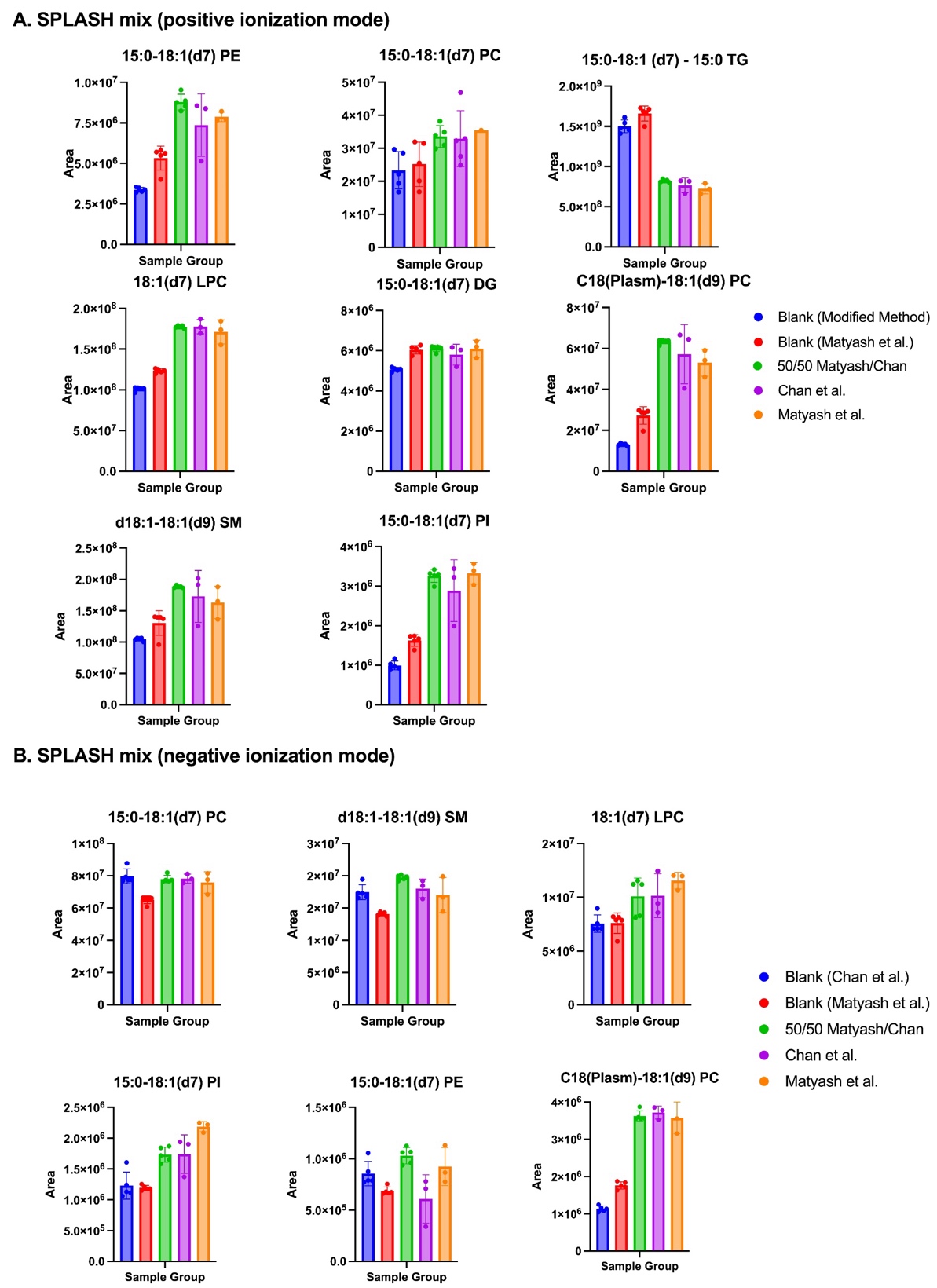


**Figure S8. SPLASH Lipidomix II internal standard comparison across different extraction methods.** The internal standard peak areas across samples processed using both the modified Chan et al. method and the Matyash et al. method, suggesting comparable extraction efficiency and minimal technical variation between methods.


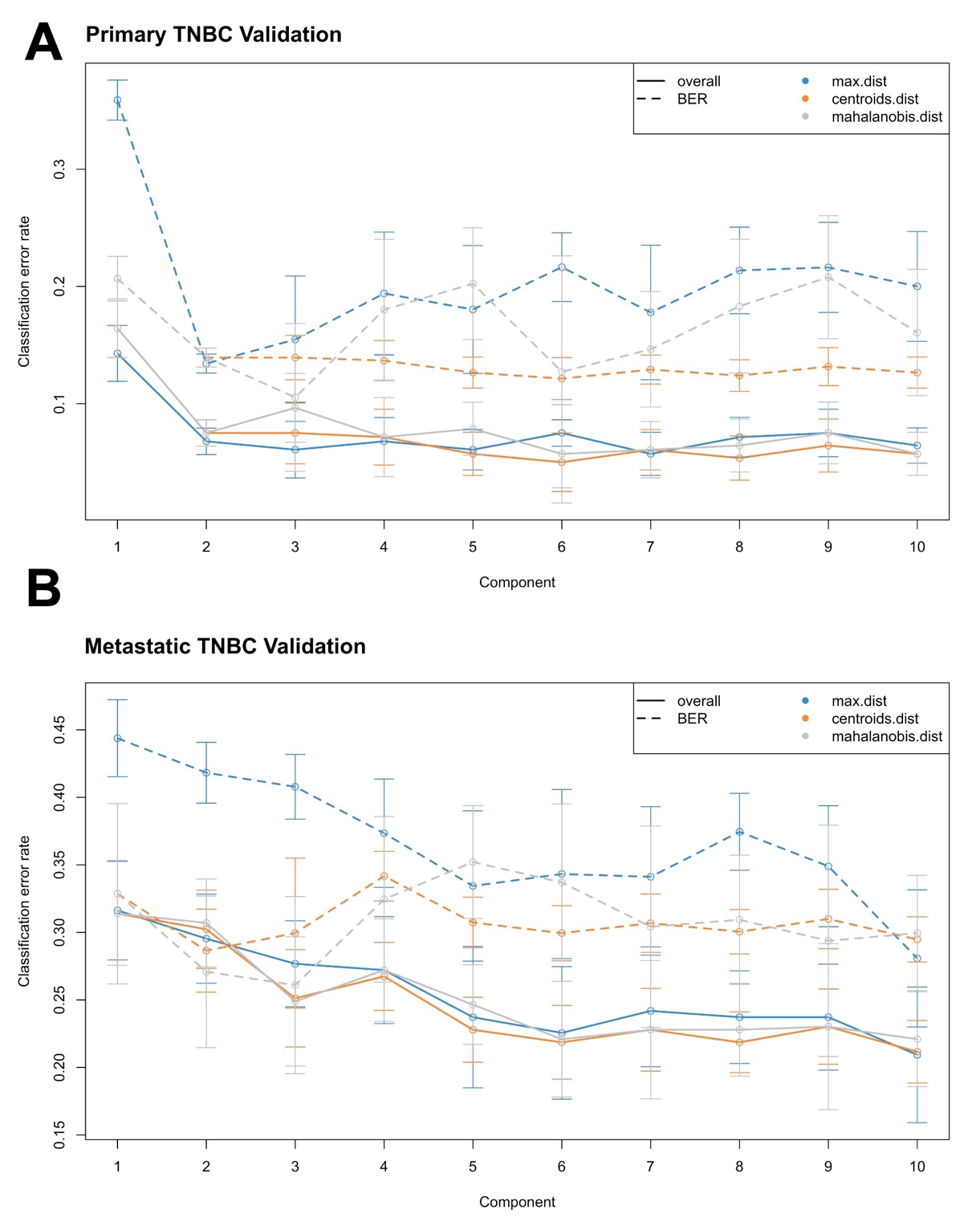


**Figure S9. PLS-DA analysis validation plots grouped by ex vivo model. (A)** Validation plot of PLS-DA analysis grouped by ex vivo models in the primary tumour. **(B)** Validation plot of PLS-DA analysis grouped by ex vivo models in the metastatic tumour.


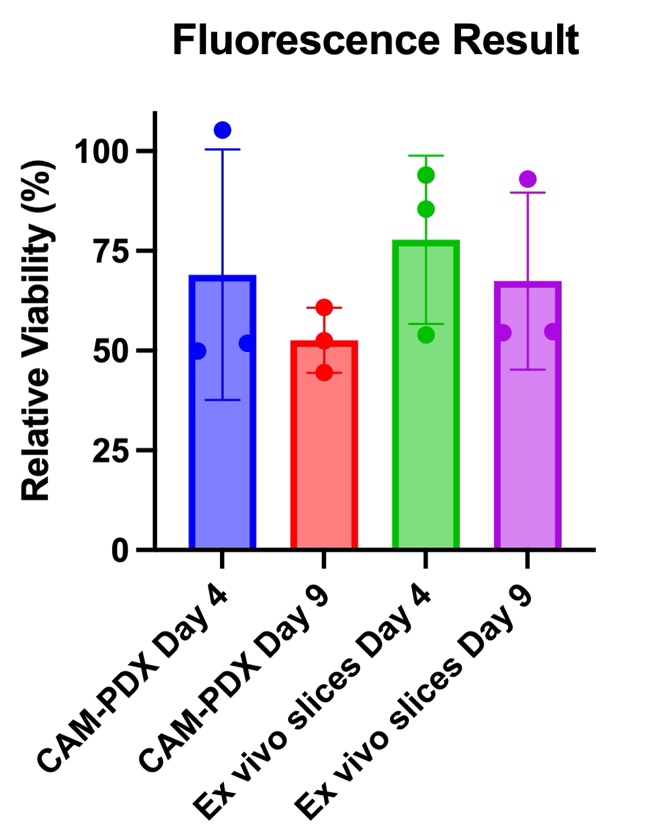


**Figure S10**. **CAM-PDX and ex vivo slices models demonstrated similar relative viability.** No statistically significant differences in viability were observed between CAM-PDX, and ex vivo slice models at Day 4 and Day 9 of the engraftment period. Fresh tumours were set as the baseline (100%) and all values were normalized accordingly. Data represent mean ± SD (n = 3).


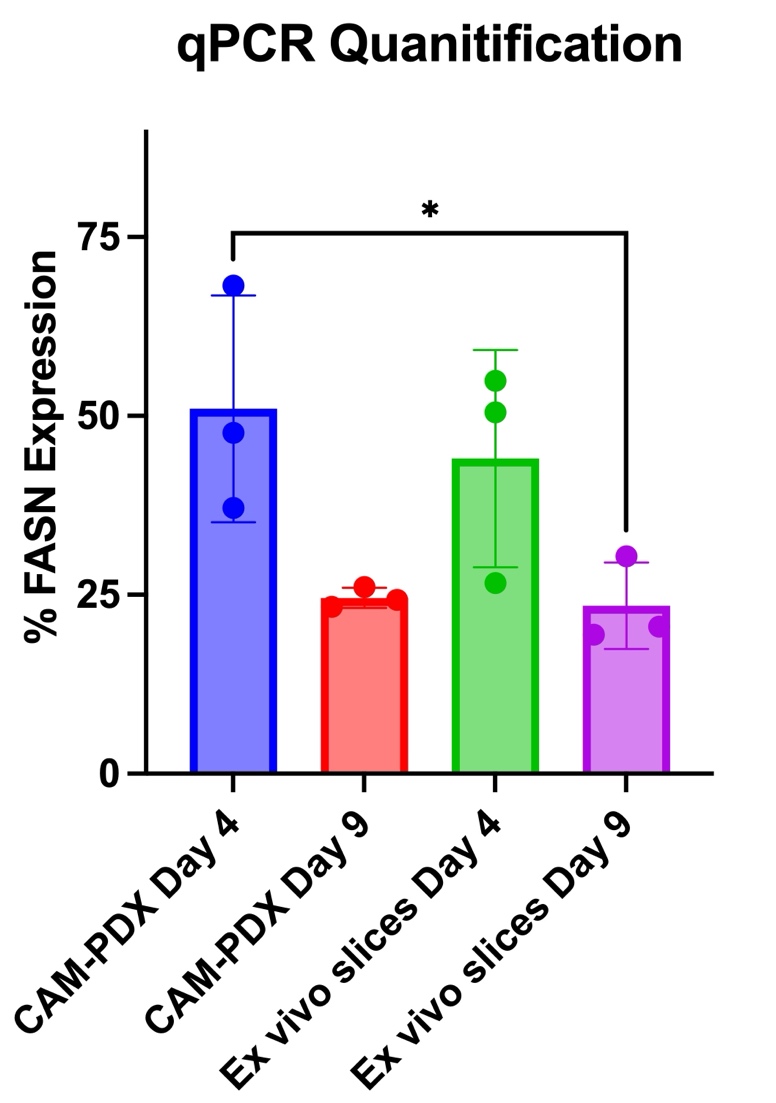


**Figure S11. FASN mRNA expression in CAM-PDX and ex vivo slices models over time**. Both ex vivo models demonstrated similar FASN expression at both time points, with day 9 samples having the lowest expression. Data represent mean ± SD (n = 3), * p < 0.05.
